# A robust strategy for overexpression of DNA polymerase from *Thermus aquaticus* using an IPTG-independent autoinduction system in a benchtop bioreactor

**DOI:** 10.1038/s41598-025-89902-4

**Published:** 2025-02-18

**Authors:** Fina Amreta Laksmi, Kenny Lischer, Yudhi Nugraha, Wiga Alif Violando, Helbert, Isa Nuryana, Firyal Nida Khasna, Naswandi Nur, Kharisma Panji Ramadhan, Destrianti Adelina Lumban Tobing, Hariyatun, Iman Hidayat

**Affiliations:** 1 Research Center for Applied Microbiology, National Research and Innovation Agency, Jalan Raya Bogor KM 46, Cibinong, Bogor, West Java 16911 Indonesia; 2 Department of Chemical Engineering, University of Indonesia, Jakarta, Indonesia; 3 Research Center for Molecular Biology Eijkman, National Research and Innovation Agency, Jalan Raya Bogor KM 46, Cibinong, Bogor, West Java 16911 Indonesia; 4 Department of Marine, Sunan Ampel State Islamic University, Surabaya, Indonesia; 5 Research Center for Ecology and Ethnobiology, National Research and Innovation Agency, Jalan Raya Bogor KM 46, Cibinong, Bogor, West Java 16911 Indonesia; 6 Research Center for Genetic Engineering, National Research and Innovation Agency, Jalan Raya Bogor KM 46, Cibinong, Bogor, West Java 16911 Indonesia; 7 Deputy for Infrastructure Research and Innovation, National Research and Innovation Agency, Jalan Raya Bogor KM 46, Cibinong, Bogor, West Java 16911 Indonesia

**Keywords:** DNA polymerase, *Thermus aquaticus*, Autoinduction, Chemically defined medium, *Escherichia coli*, DNA, Enzymes

## Abstract

The DNA polymerase derived from *Thermus aquaticus* is the most widely utilized among various DNA polymerases, indicating its significant economic importance. Consequently, efforts to achieve a substantial yield of Taq DNA polymerase (Taq-pol) are ongoing. The expression of recombinant protein using T7-induced promoters presents challenges in cost-effectiveness, primarily due to the reliance on traditional induction method. Our study aims to enhance cost-efficiency, and scalability of our method for overproducing Taq-pol, particularly in comparison to traditional IPTG-induced techniques, which remain underreported in the current literature. To achieve those purposes, this work integrated the use of (1) a high copy number vector; (2) an optimized chemically defined medium; and (3) optimized fermentation conditions in a 5 L bioreactor. A total of 83.5 mg/L of pure Taq-pol was successfully synthesized in its active form, leading to a 9.7-fold enhancement in protein yield. This was achieved by incorporating glucose, glycerol, and lactose into a defined medium at concentrations of 0.1, 0.6, and 1%, respectively, under specific production conditions in a 5 L bioreactor: 300 rpm, 2 vvm, and 10% inoculant. The data collectively suggest that the strategy serves as a significant foundation for the future advancement of large-scale production of Taq-pol.

## Introduction

Polymerase Chain Reaction (PCR) is a fundamental laboratory technique in biotechnology, utilized across various applications including genetic cloning, diagnostic instruments, sequencing, genotyping, and gene expression measurements. The heat-stable activity of Taq Polymerase Taq-pol, effective at temperatures ranging from 75 to 80 °C and resistant to inactivation during DNA double-strand denaturation in PCR, has greatly improved the potential for genetic amplification since its discovery^1–3^. The three-dimensional structure of Taq-pol is the same as that of *E. coli* pol I; however, it does not possess 3′-5′ exonuclease proofreading activity. Consequently, the amplification procedure is susceptible to errors when conducted with this polymerase^4,5^. It remains a widely favored option for PCR amplification because of its thermostability, and is also utilized in diagnostic and fundamental molecular biology studies.

Improved DNA affinity and shorter extension times have resulted from several Taq polymerase mutation investigations^6^. Meanwhile, chimeric proteins were also created to produce Taq polymerase that could proofread^7,8^. Extensive research has also been conducted on the isolation and production of recombinant DNA polymerase from various species, including hyperthermophiles^5,9^, as well as various purification techniques^10–12^. Nevertheless, research on the appropriate production of recombinant Taq polymerase at a fermenter size to improve protein yield for industrial applications has been inadequate. Thus, this study focuses on a strategy that combines a high expression vector with an autoinduction method in a 5 L fermentor to achieve the intended purpose.

Taq polymerase was synthesized under the regulation of a T7 RNA polymerase (T7RNAP)-based promoter, utilizing the codon-optimized Taq polymerase developed in a prior study^13^. A high copy number expression vector was selected to achieve a significant yield of Taq polymerase, and the plasmid copy number (PCN) following the insertion of the gene encoding Taq polymerase was assessed in this study. In addition, to enhance the production efficiency of Taq polymerase, the recombinant protein was expressed without the induction of Isopropyl ß-D-1-thiogalactopyranoside (IPTG), a costly reagent in recombinant protein production. The Taq polymerase was produced using an autoinduction system with inexpensive lactose as a natural inducer, eliminating the need for monitoring culture growth^14^.

The generation of recombinant protein in an *E. coli* system is significantly affected by medium components, pH, dissolved oxygen (DO), and physical parameters like temperature, agitation, aeration, and fermentation duration^15–18^. Consequently, the optimization of conditions for the manufacture of recombinant Taq polymerase from *Thermus aquaticus* in *E. coli* utilizing a 5 L fermenter was performed. This study illustrates that specific fermentation parameters, such as temperature, agitation speed, aeration rate, and complex media composition, affect the yield of recombinant protein production in the fermenter and contribute to the development of an efficient strategy for industrial Taq polymerase production.

## Results

### Evaluation of qPCR

Real-time quantitative PCR (qPCR) technology provides a rapid and dependable method for quantifying any target sequence present in a sample^19^. Numerous methods exist for quantifying nucleic acids, yet real-time qPCR currently stands out as the most sensitive and precise approach^20^. Earlier studies have also detailed the use of qPCR for the measurement of PCN in bacterial samples. The evaluation of qPCR as an analytical tool for PCN quantification involved an investigation into two critical parameters: specificity and efficiency. The specificity of the qPCR assay was assessed through the analysis of the melting curve, while amplification efficiency (E) serves as a crucial parameter for evaluating qPCR data analysis by scrutinizing the quantity of DNA sequences. In optimal circumstances, the quantity of DNA sequences will increase twofold in every cycle, with the percentage of E-1 reaching 100% (E equals 2)^21^. Nevertheless, factors like variations in enzymes, primers, and probes contribute to the fact that PCR efficiency seldom achieves 100%^22^. Consequently, E represents any value within the range of 1 to 2.

**Fig. 1:**
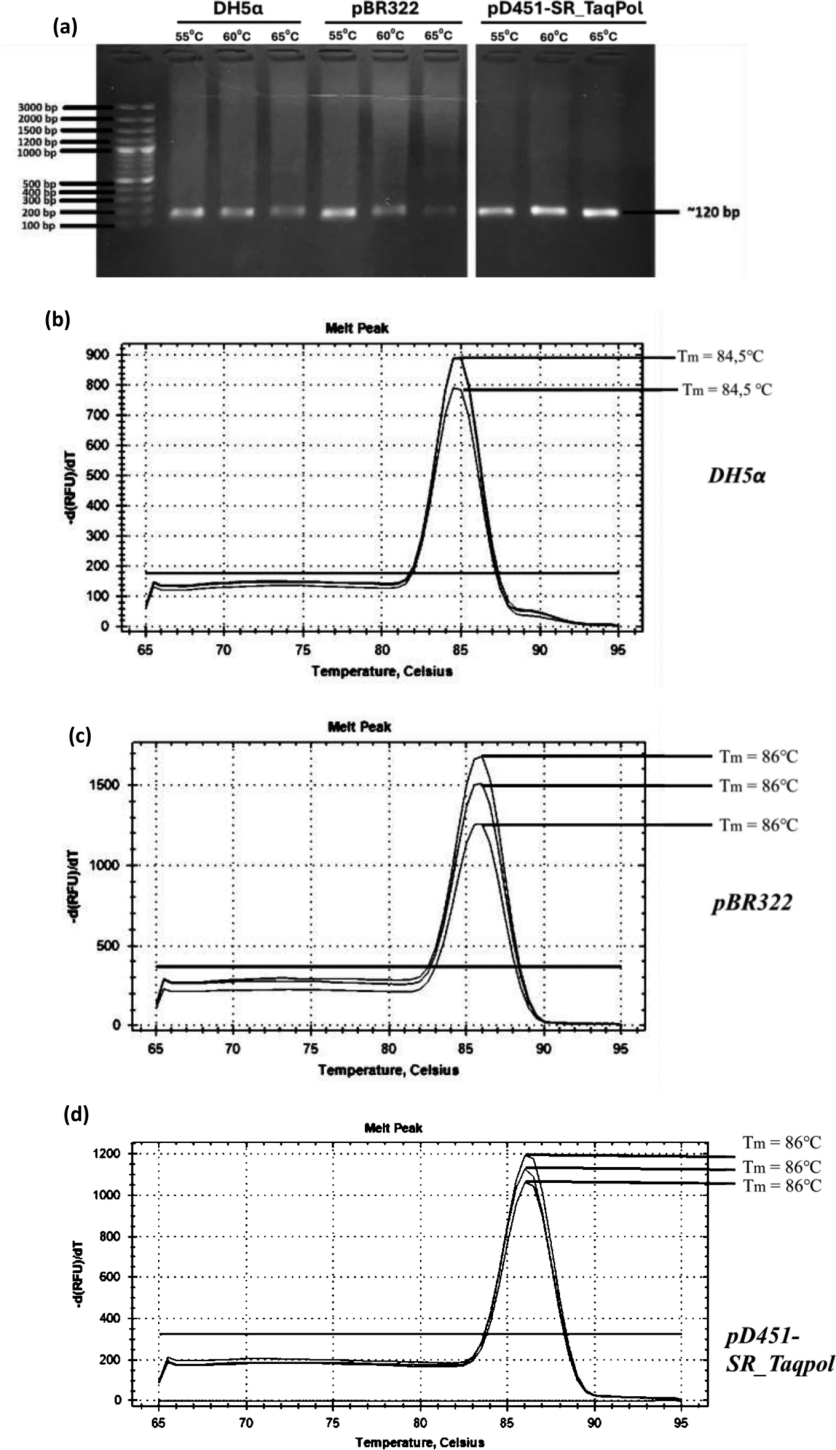

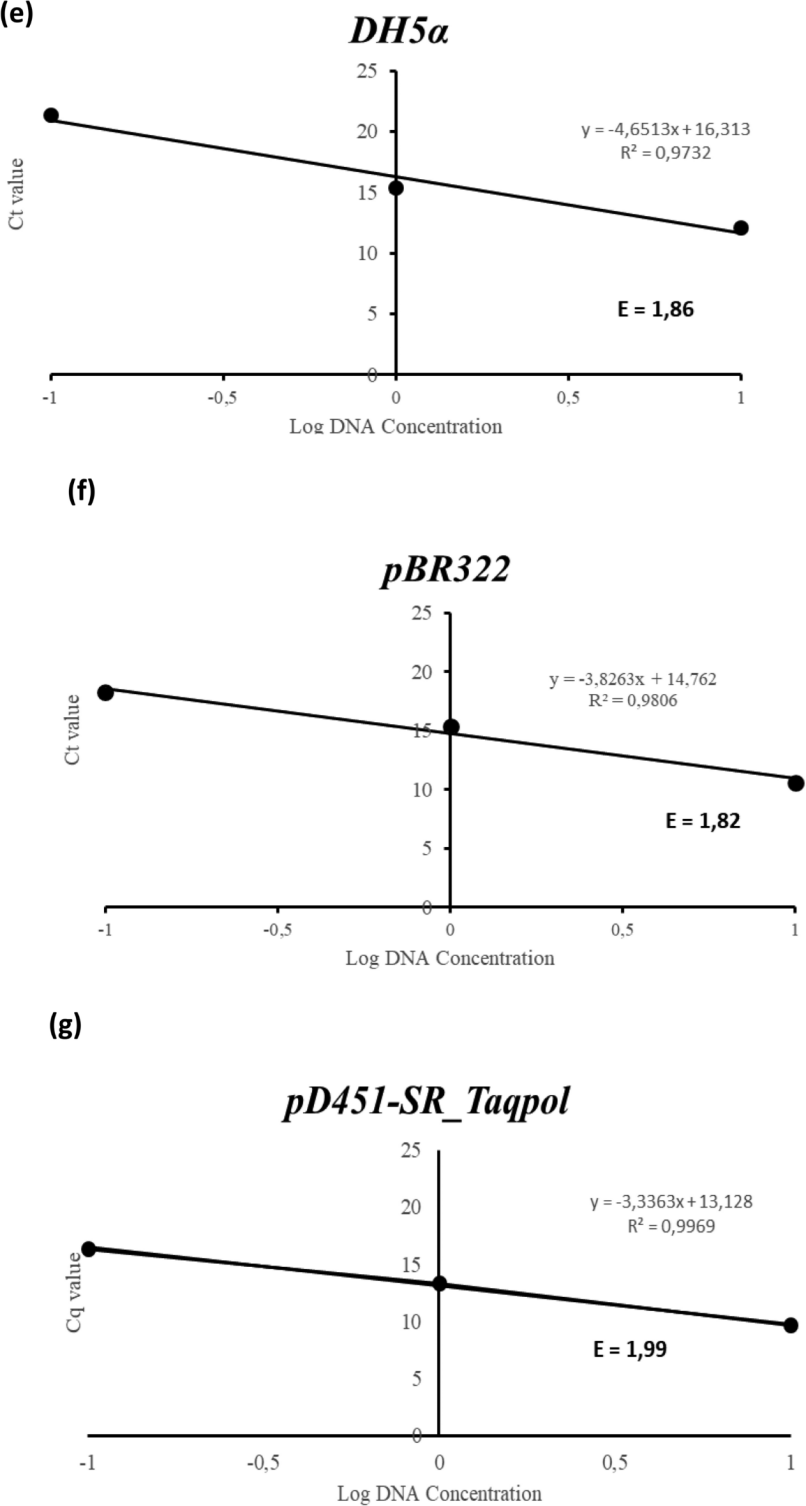
Analysis of primer specificity, efficiency, and absolute quantification. (**a**) Qualitative assessment of primer specificity for DH5α, pBR322, and pD451-SR_TaqPol at annealing temperatures of 55, 60, and 65 °C using gel electrophoresis. Quantitative assessment of primer specificity using qPCR for (**b**) DH5α, (**c**) pBR322, and (**d**) pD451-SR_TaqPol, respectively. Evaluation of primer efficiency at various DNA template concentrations for (**e**) DH5α, (**f**) pBR322, and (**g**) pD451-SR_TaqPol, respectively.

The first evaluation was on the specificity of the primers set that were utilized form amplification of gene (ori) of pD451-SR_Taqpol and pBR322, as well as DH5α gene (rrs). The primers specification was observed using qualitative and quantitative approach. The qualitative assay was conducted by performing conventional PCR using three variation of annealing temperature, which were 55, 60, and 65 °C and observing the PCR product on the electrophoresis gel. The result showed that single band was observed at 120 bp on all PCR products of three genes at all three different annealing temperature indicating that the amplification was specific (Fig. 1A). Further investigation using qPCR confirmed the amplification specificity. It was shown that one distinct peak was observed for each primer set, indicating a melting temperature of 86 °C for the gene (ori) of pD451-SR_Taqpol and pBR322 and a melting temperature of 84.5 °C for rrs gene (Fig. 1B–D). Annealing temperature of 65 °C was selected for determination of PCN using qPCR.

To assess the efficacy of the primer design against the DNA template and to identify the ideal range of DNA quantities for PCN measurement, a primer efficiency assay was conducted. An analytical range of measurement for pure DNA was determined at 10, 1, and 0.1 ng. In Fig. 1E–G, the result of the primer efficiency assay was displayed. The primer efficiency in amplifying the rrs gene was 1.86 or 86%. For amplifying the ori gene from the plasmid pBR322 and pD451-SR_Taqpol, the primer efficiency values were 1.82 or 82% and 1.94 or 94% respectively. A primer efficiency score ranging from 1.8 to 2.0 or 80–100% suggests that the amplicon product is specific^23^. Based on these findings, the primer efficiency was optimal when the amount of DNA was between 10 and 0.1 ng (Table 1).

**Table 1:** Primer sequences.

Primer	Primer types	Sequences (5′–3′)	T_m_ (°C)	Amplikon size (bp)
rrs_16SrRNA	Forward	GCAGGCGGTTTGTTAAGTCA	59.05	114
	Reverse	TCACCGCTACACCTGGAATT	59.02	
ori_pBR322	Forward	AACCGCGTAAGACACGACTT	58.96	105
	Reverse	AGGCCACCACTTCAAGAACT	59.15	
ori_pD451	Forward	AGTGTAGCCGTAGTTAGGCC	58.89	120
	Reverse	AACCCGGTAAGACACGACTT	58.96	

### PCN determination of pD451-SR_Taqpol

Supplementary Fig. S1. displayed the standard curves for target (ori) and reference (rrs) gene, which range from 1 × 10^6^ to 1 × 10^10^ copies/μl. Both curves are linear within the tested range, with an R^2^ value greater than 0.999. The standard curves for ori and rrs had slopes of − 3.452 and − 3.505, respectively, representing similar values. The same slope indicated that the evaluation of the reference gene and target gene is 1:1, which was desirable^24^. Simultaneous qPCR amplifications were conducted on the DNA samples from pBR322 and pD451-SR_Taqpol and the CT values were used to calculate the plasmid copy number of pBR322 and pD451-SR_Taqpol from standard curves. The findings from the absolute quantification along with the determined plasmid copy numbers are presented in Table 2. The analysis result indicated that pBR322 possesses a copy number of approximately 22.38 copies per cell, in contrast to pD451-SR_Taqpol, which has a copy number of 77.62 copies per cell.

**Table 2:** Absolute determination of PCN.

Sample	Ct values^a^		Copy number (copies/cell)^b^		PCN^c^
	ori	rrs	ori	rrs	
pBR322 (standard)	10.98 ± 0.07	16.39 ± 0.13	2.3 × 10^8^	1.0 × 10^7^	22.38 (4.8%)
pD451-SR_Taqpol	9.11 ± 0.00	16.39 ± 0.13	8.3 × 10^8^	1.0 × 10^7^	77.62 (8.1%)

^a^Mean ± S.D. (n = 2).

^b^Mean (coefficient) (n = 2).

^c^Mean (coefficient) (n = 2).

### Expression of Taq-pol via autoinduction

**Fig. 2:**
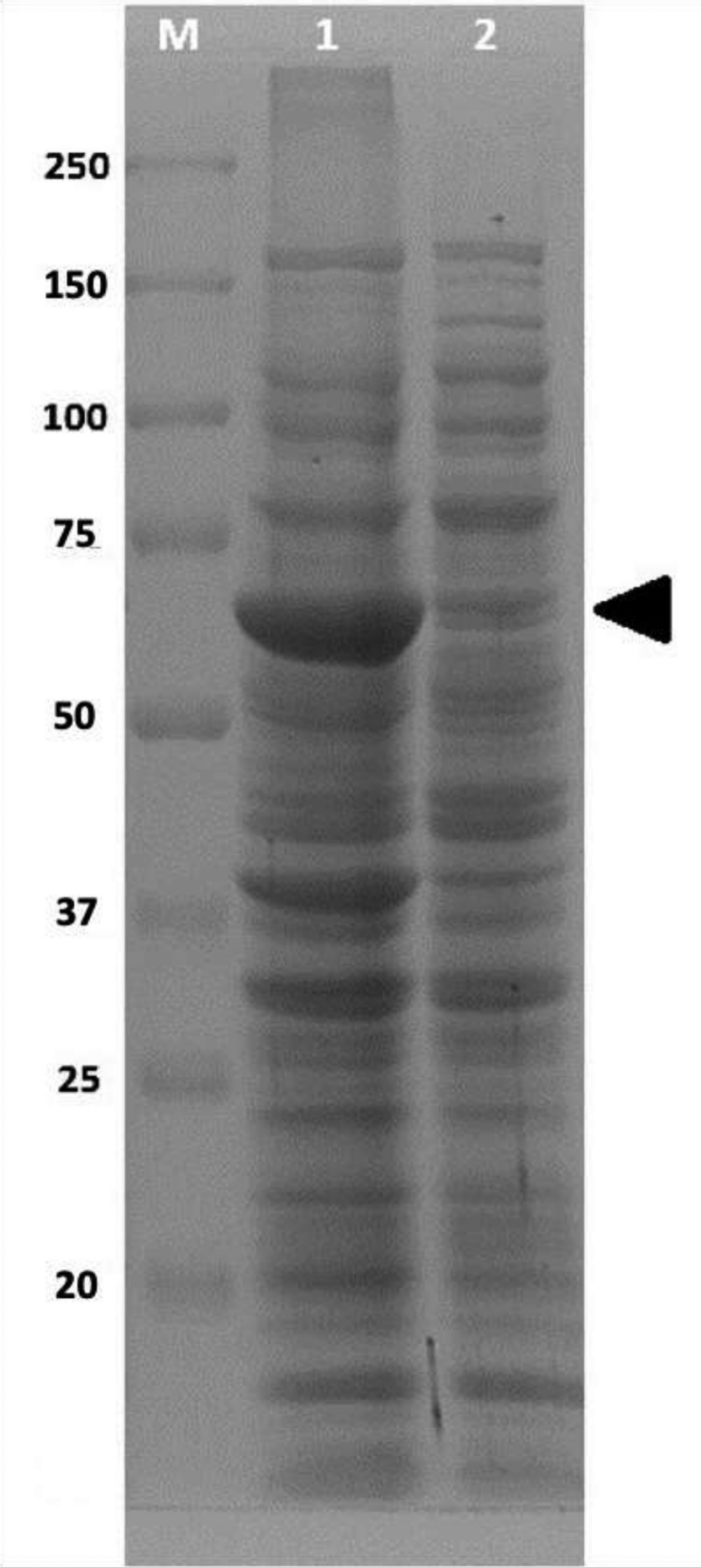
SDS PAGE analysis of Taq-pol expression using autoinduction method in chemically defined medium. The triangular sign denoted the target protein band. Lane M: protein marker (Precision Plus protein ladder, Biorad (German)); lane 1: a Taq-pol soluble fraction synthesized by autoinduction; lane 2: negative control.

**Fig. 3:**
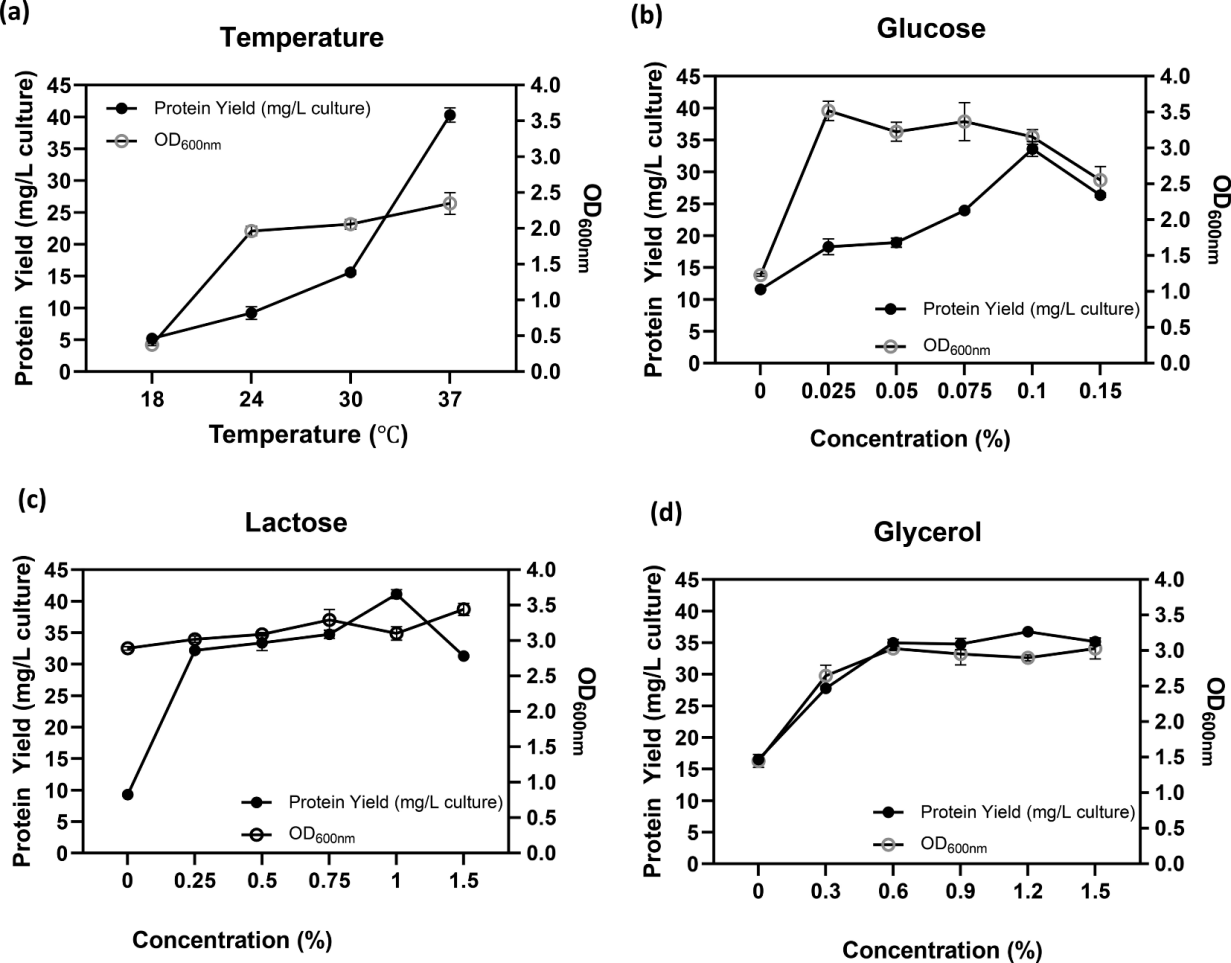
Impact of Temperature and Carbon Source on Taq Polymerase Production. (**a**) Effect of different temperature conditions on Taq Polymerase yield. (**b**) Effect of glucose, (**c**) lactose, and (**d**) glycerol as carbon substrates on protein production. Data represent the mean of triplicates ± standard deviation, with black dots (•) indicating protein yield (g/L culture) and light dots (○) indicating OD_600nm_ values.

Autoinduction was facilitated by cultivating *E. coli* BL21 star™ (DE3) carrying pD451-SR_Taqpol at 37 °C in a chemically defined medium supplemented with 0.5% lactose for 24 h. According to the prior study that used a conventional induction approach, the molecular mass of recombinant Taq-pol was estimated to be around 63 kDa^13^. To verify the expression of recombinant Taq-pol via the autoinduction method, *E. coli* BL21 star (DE3) lacking the pD451-SR_Taqpol insertion served as a negative control and was cultivated under similar conditions to the recombinant cells. Following sonication, the supernatant of the sample and the negative control were utilized for SDS-PAGE analysis. Analysis of SDS-PAGE revealed a prominent band at approximately 63 kDa corresponding to the soluble form, while no overexpressed protein of similar size was observed in the negative control. This indicates successful overexpression of recombinant Taq-pol using the autoinduction technique in a chemically defined medium (Fig. 2). The incubation temperature significantly influences the metabolism and growth of recombinant *E. coli*. Consequently, the recombinant Taq-pol was optimized for incubation at temperatures of 18, 24, 30, and 37 °C for a duration of 24 h (Fig. 3A). The results indicate that the highest growth of recombinant *E. coli* was achieved at 37 °C and the fast-growing recombinant *E. coli* did not affect the solubility of Taq-pol.

### Optimization of carbon source

The optimal concentrations of glucose, lactose, and glycerol in the chemically defined medium were investigated to enhance the production of recombinant Taq-pol. The yield of recombinant Taq-pol increased with the concentration of glucose, ranging from 0 to 0.1%, as illustrated in Fig. 3B. However, the protein yield diminished as the glucose concentration increased further. Figure 3C illustrates the impact of lactose content on the production of recombinant Taq-pol. The increase in recombinant Taq-pol yield was clearly mediated by a lactose raise of up to 1%. Protein production was adversely affected by concentrations exceeding 1%. The influence of glycerol concentration on Taq-pol synthesis was analyzed (Fig. 3D). Glycerol concentrations of up to 0.6% were demonstrated to enhance the production of recombinant Taq-pol. Yields of protein were equivalent at elevated concentrations.

### Optimization of fermentation condition using a 5 L bioreactor

**Fig. 4:**
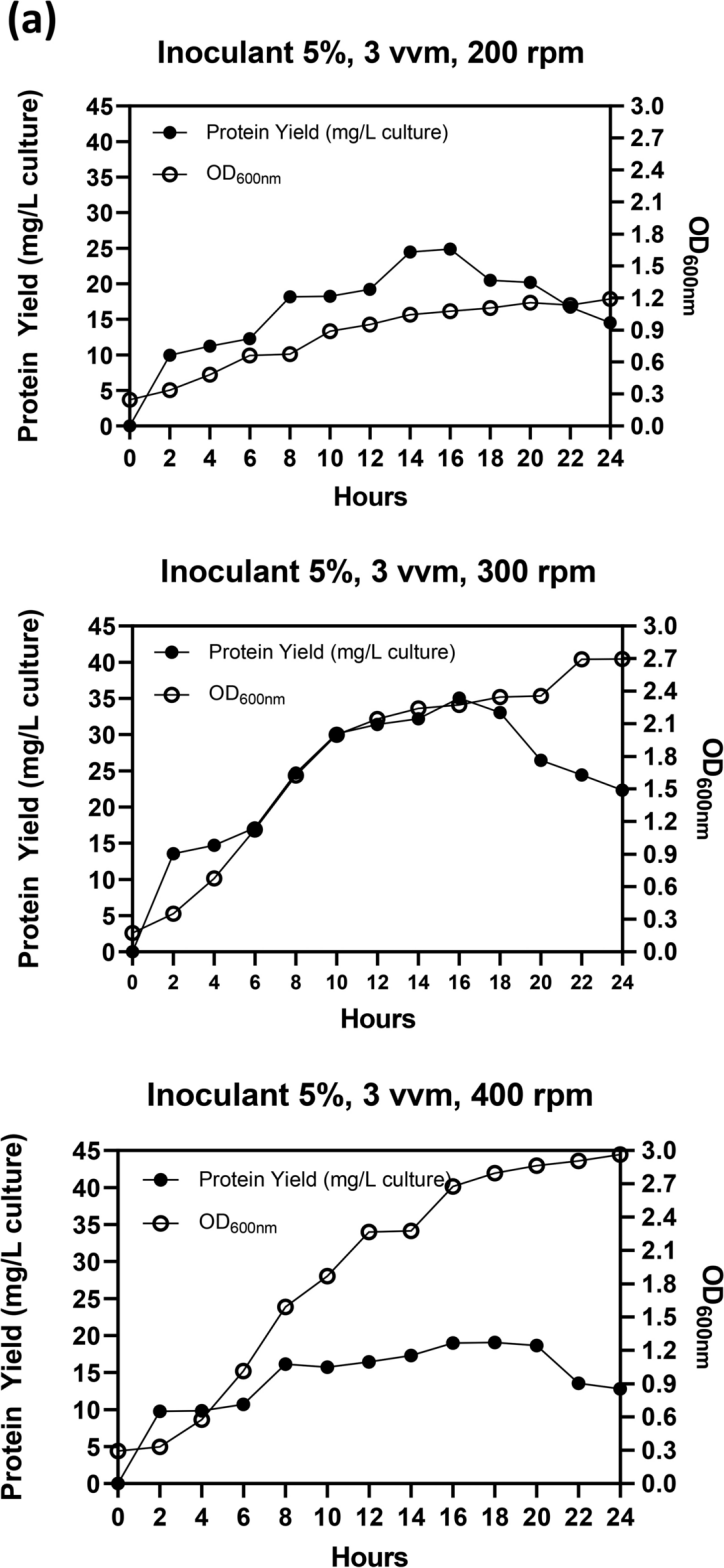

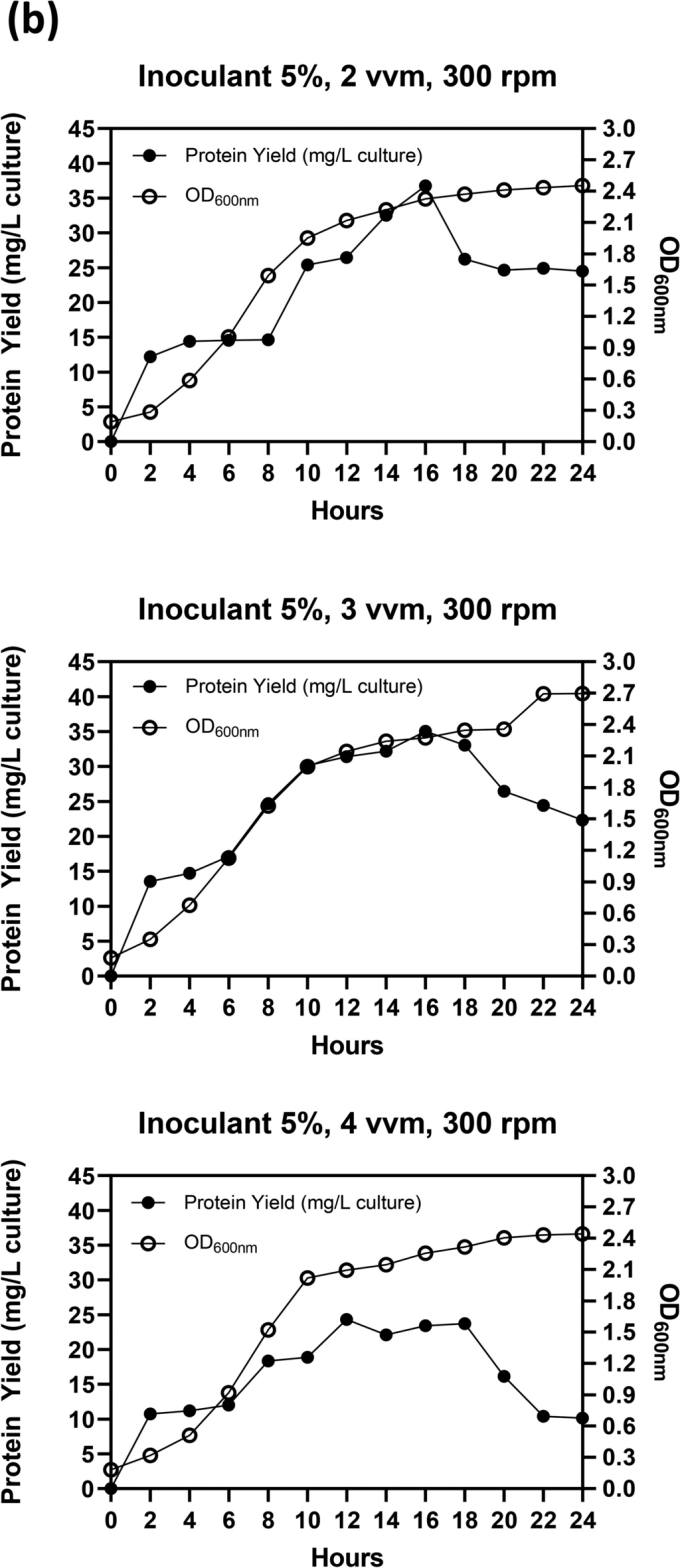

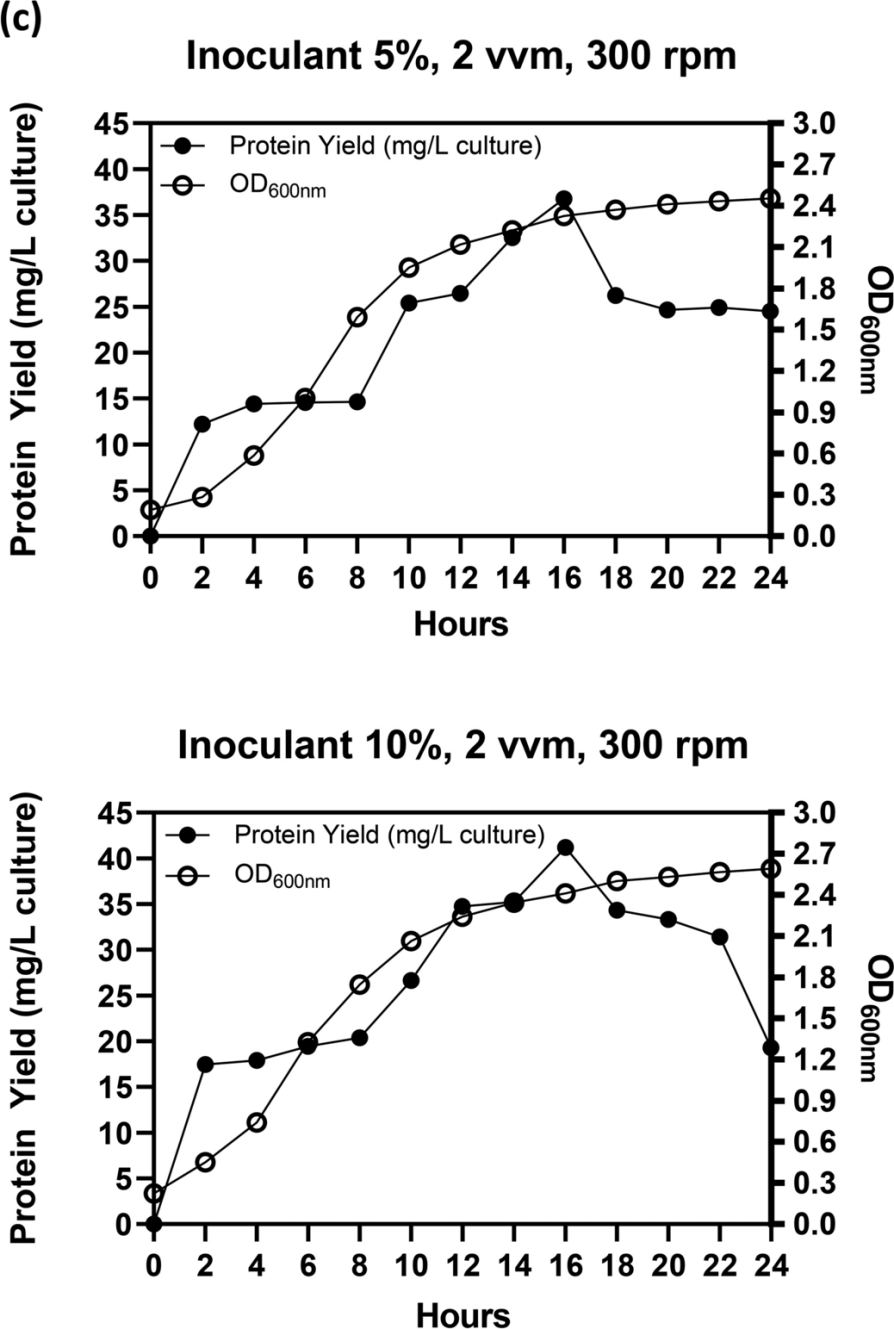
Optimal condition of aeration and agitation on recombinant Taq-pol production in a 5 L Bioreactor (3 L Working Volume) using autoinduction method in chemically defined medium. (**a**) Effect of agitation speeds at 200, 300, and 400 rpm over a 24-h cultivation period. (**b**) Effect of aeration rates at 2, 3, and 4 vvm under constant agitation for 24 h. (**c**) Effect of inoculum concentrations at 5% and 10%. Black dots (•) represent protein yield (g/L culture), while light dots (○) indicate OD600nm values.

Following optimization at the flask scale, the parameters related to agitation, aeration, and inoculant were further evaluated using a 5 L bioreactor. The initial endeavor to enhance the production conditions of Taq-pol was adjusting the agitation while maintaining aeration at 3 vvm with a 5% inoculant. Figure 4A illustrates that a positive trend was seen in both Taq-pol production and microbial biomass by increasing agitation speeds by up to 300 rpm, especially after 2 h of incubation. This analogous tendency was also evident in the microbial biomass when 400 rpm was utilized. Nonetheless, boosting the agitation to 400 rpm led to a reduction in protein yield. Consequently, 300 rpm was determined to be the optimal environment for the production of Taq-pol. The subsequent attempt involved the use of a variety of aeration. Figure 4B demonstrated that a comparable trend was observed in both Taq-pol yield and microbial biomass at aeration of 2 and 3 vvm. However, a similar tendency was not observed when the aeration was raised to 4 vvm. Even though microbial biomass displayed a comparable trend to the prior aeration condition, a reduction in protein yield was observed; hence, 2 vvm was selected for subsequent application.

**Fig. 5:**
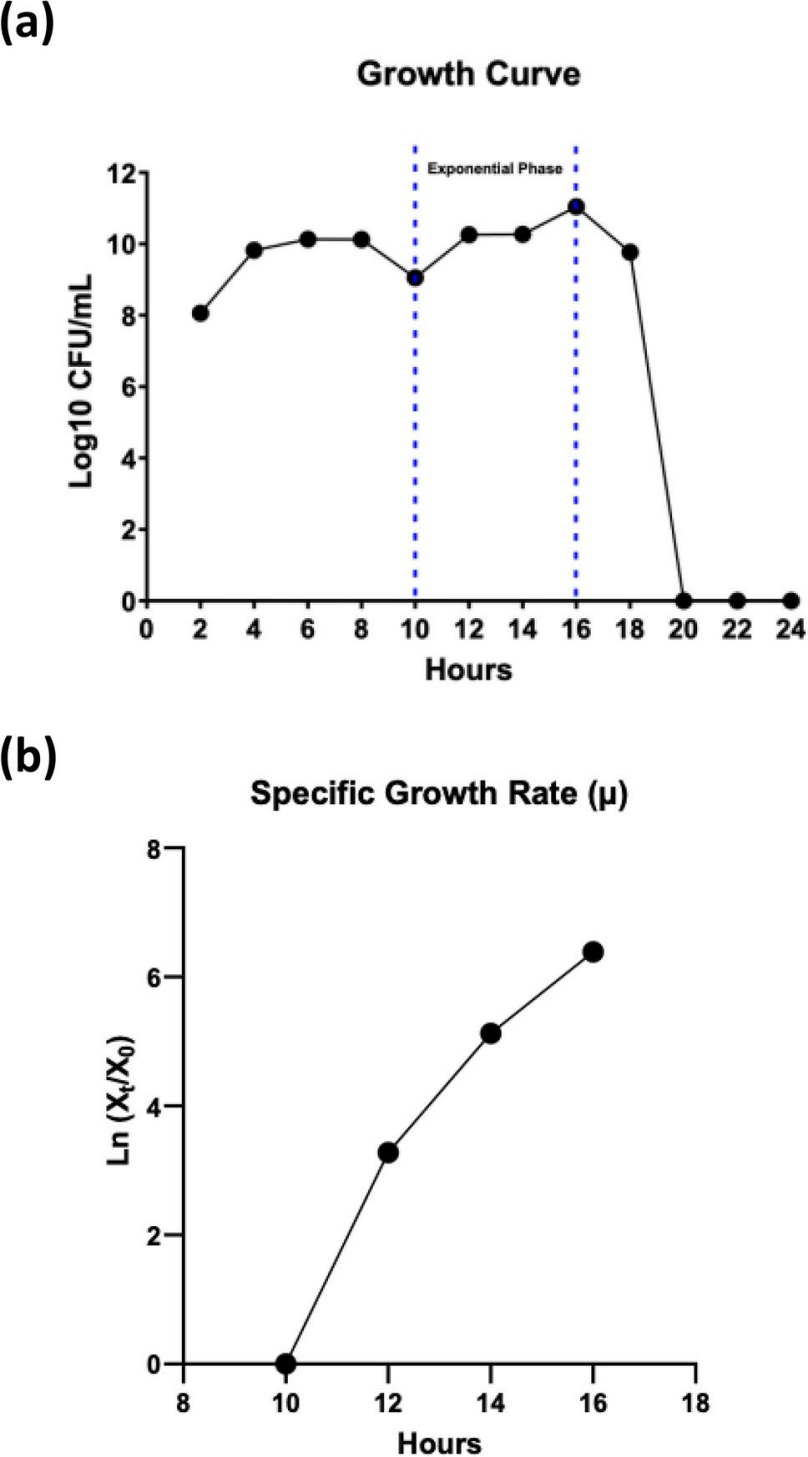
Growth curve and specific growth rate of recombinant Taq-pol in *E. coli* BL21 Star (DE3). (**a**) the growth profile and (**b**) specific growth rate of *E. coli* BL21 star^TM^ (DE3) during the production of recombinant Taq-pol, highlighting the dynamics of cell growth over time.

A variation of 5 and 10% of inoculant was transferred to the culture to examine its effect (Fig. 4C). The investigation demonstrated that higher Taq-pol production was observed with the addition of 10% inoculant compared to the use of 5% inoculant. This finding indicates that a high cell density inoculum is necessary to achieve a beneficial impact on microbial growth. The microbial growth during fermentation was analyzed, and the findings are illustrated in Fig. 5. The exponential phase commenced after 10 h of incubation. The peak growth of approximately 8.84 × 10^11^ CFU/mL was attained after 16 h of incubation, corresponding to a maximum specific growth rate of 0.7976 μmax/h.

### Purification and activity assessment of recombinant Taq-pol

**Fig. 6:**
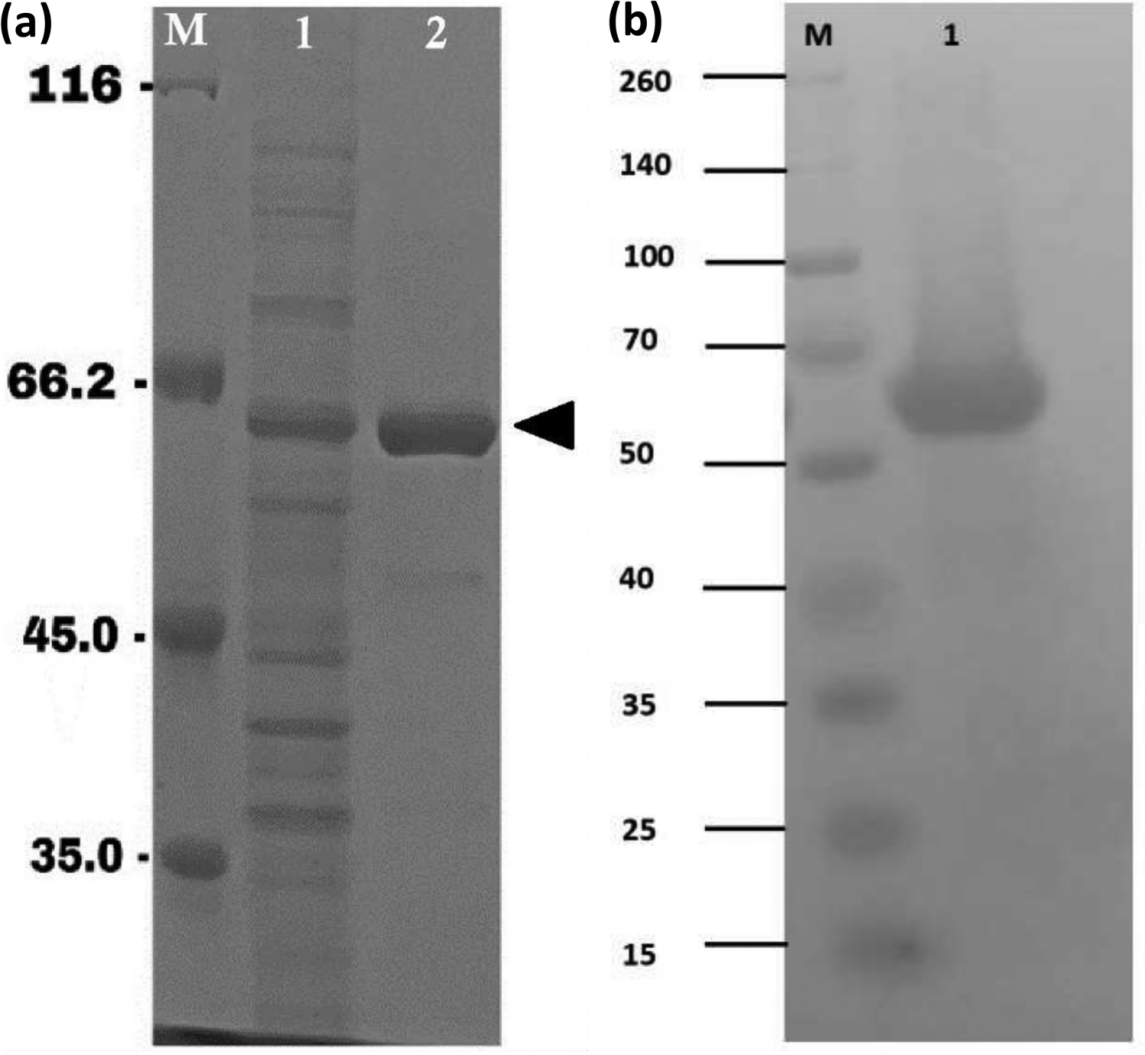
Assessment of pure Taq-pol using SDS-PAGE and western blot. (**a**) SDS-PAGE analysis of purified Taq-pol from small-scale culture. Lane M: protein ladder (Pierce™ Unstained Protein ladder, Thermo Fisher Scientific (USA)); lane 1: crude Taq-pol; lane 2: purified Taq-pol. The solid triangle indicates the target protein at 63 kDa. (**b**) Western blot analysis. Lane 1: purified Taq-pol; lane M: Protein ladder (Spectra Multicolor Broad Range Protein Ladder, Thermo Fisher Scientific (USA)).

Following the identification of the optimal conditions, the production of Taq-pol was carried out in a 5 L bioreactor using those established parameters. The purity of theTaq-pol was subsequently attained following the one-step purification process utilizing the HisTrap™ HP column. The purified Taq-pol was successfully obtained and subsequently utilized for activity assessment through a qPCR method. The effectiveness of the purification process of the enzyme can be assessed by observing its specific activity. The data presented in Table 3 illustrates that the increase in specific activity of the purified Taq-pol signifies a rise in the purification fold. The purified Taq-pol yield was determined to be approximately 25.86%, achieving 4.55-fold purification. The total protein concentration and purified Taq-pol levels were quantified using the BCA assay, resulting in values of 935 mg/L for the culture and 83.5 mg/L for the purified enzyme, respectively. Additionally, both crude and purified Taq-pol underwent SDS-PAGE (Fig. 6A) and western blot analysis (Fig. 6B). The analysis indicates that the recombinant Taq-pol was effectively expressed and purified, displaying a protein band consistent with the previous report^13^ of 63 kDa.

**Table 3:** Purification of recombinant Taq-pol.

Process	Total activity (U)	Total protein (mg/100 mL culture)	Specific activity (U/mg)	Purification (fold)	Yield (%)
Crude Enzyme	15,200	187	40.64	1	100
HisTrap HP	4010	16.71	185.04	4.55	25.86

## Discussion

Plasmids serve as important tools for the introduction of foreign genes into recombinant cells. Plasmids exist in multiple copies within cells, and the quantity of a plasmid is a crucial determinant of gene expression^25^. An elevation in the production of recombinant protein is expected from transformants with high copy number due to the gene dosage effect^26^. Numerous plasmids with high copy number have been engineered and effectively utilized for elevated protein expression in *E. coli* system^27–29^. Moreover, the stability of plasmids is crucial in fermentation processes involving recombinant systems, as it ensures the longevity and expression of exogenous genes^30^. Plasmid stability provides an invaluable understanding of the essential properties of cells throughout the processes of recombinant protein production ^31^. Hence, a thorough evaluation of plasmid stability is critical for the dependable production of recombinant protein. In this study, a plasmid copy number (PCN) assay is employed for quantitative investigation of plasmid stability. During log phase growth conditions, the PCN value of pBR322 was 22 copies, aligned with the reference, falling within the range of 15–32 copies^24^. This suggests that PCN quantification is sufficiently accurate to determine the PCN value of the pD451-SR_Taqpol plasmid for Taq-pol production. The result of PCN determination confirmed that the pD451-SR_Taqpol plasmid used in this study had a high copy number^32,33^.

The implementation of an autoinduction method for production of recombinant protein in the *E. coli* system overcomes the limitations of traditional induction methods, thereby rendering it more suitable for pilot-level fermentation. The autoinduction technique for protein expression leverages the regulatory components of the lac operon, including glucose, glycerol, and lactose, during two distinct phases of cellular growth. This process facilitates a seamless transition from the uninduced to the induced state, all meticulously governed by the expression host. Initially, glucose is consumed until it is fully depleted, after which the focus shifts to the utilization of lactose and glycerol in the induced state. The reduction of glucose triggers the release of catabolite repression, leading the way for lactose to serve as an inducer, while glycerol supports cell growth^14,34^.

Overexpression of functionally active recombinant reteplase^35^, silk-elastin-like polymers, isoallergen tropomyosin, and pullulanase were reported by utilizing autoinduction method. A recent report demonstrated the successful application of the autoinduction method using a chemically defined medium for producing reverse transcriptase^17^ and DNA polymerase from *Pyrococcus furiosus*^18^, highlighting the potential of this approach for synthesizing Taq-pol. The application of a chemically defined medium minimize the significant differences in the composition of media, particularly concerning the levels of yeast extract and peptone^36,37^. The composition of yeast extract and peptone may differ considerably due to variations in yeast strains, sources of peptone, autolysis methods, production processes, or subsequent purification techniques^37^. Therefore, employing a chemically defined medium with precise control can mitigate these variations in the production of recombinant Taq-pol.

The strategy for autoinduction in protein production relies on the regulatory aspects of the lac operon, that involve glycerol, glucose and lactose, across two phases of cellular growth^34,38^. This process enables the transition to an induced state, which is entirely orchestrated by the expression host, and thus eliminating the need for monitoring the growth of the host. While the standard autoinduction media included 0.05% glucose, 0.5% glycerol, and 0.2% lactose^14^, the concentrations were noted to vary based on the specific protein being synthesized. Following optimization, the optimal medium concentrations for glycerol, glucose and lactose, were found to be 0.6, 0.1, and 1%, respectively, yielding the maximum Taq-pol output.

Physical parameters, such as aeration and agitation, have a substantial impact on the proliferation of microorganisms and the generation of their metabolic products in bioreactors^39–41^. Fermentation medium is efficiently heated, oxygenated, and nutrient-transferred by the use of agitation^42^. Increased agitation velocities lead to greater power consumption and produce uneven shear and mixing pressures, which can adversely affect host cells and hinder protein production ^43^. Conversely, insufficient velocity of agitation will increase the viscosity of the fermentation medium, leading to reduced mass transfer efficiency^44^. Aeration fulfills two critical functions: it supplies necessary oxygen for cellular growth and facilitates the removal of effluent gas generated during the fermentation process^42^. Consequently, it is essential to optimize both aeration and agitation for the production of Taq-pol in a pilot-scale fermentation process. In this study, the results indicated that the aeration and agitation of about 2 vvm and 300 rpm, respectively, were adequate to achieve the maximum yield of Taq-pol.

**Fig. 7:**
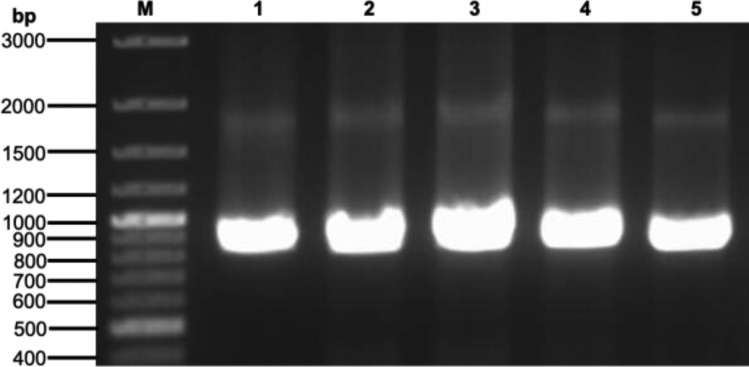
Qualitative assessment of Taq-pol activity. Lane 1, 0.6 U commercial Taq-pol; Lane 2, 1.25 U purified Taq-pol; Lane 3, 0.63 U purified Taq DNA polymerase; Lane 4, 0.31 U purified Taq-pol; Lane 5, 0.15 U purified Taq-pol.

In the earlier work, we used an induction method to produce 8.5 mg/L of purified Taq-pol^18^. Nevertheless, Taq-pol yield was significantly enhanced using the methodology employed in this study. The concentration of pure recombinant Taq-pol was 83.5 mg/L, representing a 9.8-fold increase over the protein target attained with the previous method. Additionally, in comparison to similar studies, our results were advantageous. Our method achieved a yield increase of up to 1.7-fold compared to the induction technique suggested by Mishra et al.^45^, which produced a notable yield of Taq-pol at approximately 50 mg/L. The auto-induction method employed in this study not only enhances yield but also provides a cost-effective alternative to the IPTG-induced method. Furthermore, prior research indicates that employing auto-induction medium for the production of recombinant proteins significantly reduces both time and cost. The estimated media costs for producing an equivalent amount of protein via an auto-induction system are 50% of the costs linked to reverse transcriptase (RT)^46^, 25% for PfSHMT^47^, 20% for PvSHMT^47^, and 5% for medium-chain fatty acid (MCFA)^48^, relative to LB-IPTG costs. The capabilities of the Taq polymerase developed in this study were further supported by the observation that it showed performance on par with the commercial Taq polymerase (Fig. 7). Consequently, this study lays forth a strategy for producing Taq polymerase that is both practical and economical for commercial use.

## Conclusion

A combination strategy utilizing a high expression vector and autoinduction method yielded a high efficiency production of approximately 83.5 mg/L culture of active Taq-pol in a chemically defined medium containing 0.6% glycerol, 0.1% glucose, and 1% lactose. This was achieved under optimized conditions in a 5 L bioreactor, with parameters set at 300 rpm, 2 vvm, and 10% inoculant. The optimized defined medium and parameters for production in a 5 L bioreactor resulted in an increase of Taq-pol by up to 9.8-fold. Furthermore, the purified Taq-pol demonstrated activity comparable to that of the commercial. This study outlines an effective method for the overproduction of active Taq-pol, rendering it appropriate for industrial use.

## Materials and methods

### Bacterial strain and plasmid

A codon-optimized gene encoding Taq polymerase^13^ inserted into plasmid pD451-SR (pD451-SR_Taqpol) was synthesized by ATUM,Inc (Newark, CA) and then cloned to *Escherichia coli* DH5α for cloning and maintaining purpose. The pD451-SR featured pUC ori allowing high copy number of plasmid per cell. In addition, the plasmid utilized T7 RNA polymerase (T7RNAP) and a gene encoding kanamycin resistance. Plasmid pBR322 (NEB, Biolabs) was selected as control for determination of PCN. The *E. coli* BL21 star^TM^ (DE3) (Invitrogen, USA) was chosen as the host expression for the production of Taq polymerase.

### Determination of plasmid copy number (PCN)

#### qPCR primer design and qPCR DNA templates preparation

The two primer set (Table 1) designed for PCN determination targeting the origin of replication gene (ori) of pD451-SR_Taqpol and pBR322. A primer set, namely rrs_16SrRNA, targeting chromosomal DH5α gene (rrs) was designed for amplification of rrs gene. All of primers were constructed using the web-based program Primer3.

Calibration of the PCN calculation necessitates the utilization of a calibrator plasmid. The rrs gene is derived from bacterial chromosomal DNA of *E. coli* DH5α, which was isolated using Quick-DNA Fungal/Bacterial Microprep Kit (Zymo, USA) to extract the genomic region. The traditional PCR method was employed to amplify the rrs gene using the rrs_16SrRNA primer set, as shown in Table 1. The reaction mixture of PCR contained 1 × Q5 Reaction Buffer, 200 μM deoxynucleotide (dNTP), 0.5 μM of each primer, 1 U Q5 Hot Start DNA Polymerase (NEB, UK), and 10 ng *E. coli* DH5α genomic DNA template. The PCR amplification procedure was conducted in accordance with the following protocol: an initial denaturation step at 98 °C for 1 min, followed by 30 cycles consisting of 10 s at 98 °C, 30 s at 64 °C, and 20 s at 72 °C. The amplification was then extended for 2 min at 72 °C. The PCR result underwent purification using the NucleoSpin Gen and PCR Clean-up (Takara, Japan) and was subsequently cloned into a pGEM-T Easy vector (Promega). The pGEM-T plasmid construct already includes the ori plasmid gene (Pori), so requiring only the ligation of the rrs gene into the plasmid. *E. coli* DH5α harboring pD451-SR_Taqpol, pBR322, and pGEM-T_rrs were cultured at 37 °C with a shaking rate of 160 rpm and the cell was harvested at mid-log phase when the optical density at 600 nm achieved 0.5. The plasmid pD451-SR_Taqpol, pBR322, and pGEM-T_rrs were isolated using Qiaprep Spin Miniprep kit (Qiagen, German).

#### Analysis of primer specification and efficiency

A variation of temperature (55, 60, dan 65 °C) was applied for determination of optimum annealing temperature. The ori gene of pD451-SR_Taqpol and pBR322 as well as the rrs gene were synthesized by conventional PCR using 10 ng DNA template, 1 × DreamTaq Green PCR Master Mix (ThermoFisher, USA) and 1 mM of two complementary primers. A PCR cycle consisting of 95 °C for 3 min, followed by 30 cycles of 95 °C for 30 s, a variation of melting temperature for 30 s, and 72 °C for 30 s, and a cycle of 72 °C for 10 min were utilized to amplify the DNA templates. The amplified DNA was subjected to electrophoresis on a 2% agarose gel, followed by visualization using a UV transilluminator. For further confirmation of the primer specification, real-time quantitative PCR (qPCR) was conducted using CFX Connect Real Time PCR (Biorad, USA). The assessment of primer specificity was conducted by analysing the melting curve of the qPCR product. The qPCR reaction mixture consisted of 1 × Toyobo SYBR Green Realtime PCR Master Mix, 0.8 μM primer pair, and 10 ng template DNA. The cycling conditions for the PCR reaction were as follows: 1 min at 95 °C, followed by 30 cycles of 15 s at 95 °C, 15 s at a varied melting temperature, and 45 s at 72 °C for all amplicons. Additionally, analysis of primer efficiency was employed to determine the optimal DNA range utilized for qPCR. The present study examines the DNA quantities of DH5α, pBR322, and pD451-SR_MMLV-RT samples range from 10, 1, and 0.1 ng. The calculation of the primary efficiency value (E) is determined by employing the method proposed by Vogels et al.^49^. In this equation, the value is derived from the linear equation curve that correlates the log template DNA with Ct values, resulting in a linear equation of the form y = Ax + B, where y denotes the Ct value, x indicates the log copy number result and A indicates the coefficient value.

$$E = 10^{{ - \frac{1}{A}}}$$

#### Construction of PCN standard curve

To establish a PCN standard curve, the plasmid pGEM-rrs was subjected to dilution using several amounts of DNA, specifically 100, 10, 1, 0.1, and 0.01 ng. Each concentration corresponds to the logarithm of the number of copies on the standard curve. The determination of the range of log copy numbers on the standard curve is based on the formula proposed by Whelan et al.^50^:

$$\frac{{6.02 \times 10^{23} \left( {{\text{copy}}/{\text{mol}}} \right) \times DNA\;amount \left( {{\text{ng}}} \right)}}{{DNA\;length \left( {{\text{bp}}} \right) \times 660\;{\text{g}} /{\text{mol}} /{\text{bp}}}}$$

The qPCR reaction mixture included 1 × Biorad SsoAdvanced Universal SYBR Green Supermix, 25 μM primer pair of rrs_16S rRNA or ori_pBR322, and a serial dilution of 0.01–100 ng of template DNA. The parameters for the PCR reaction were established as follows: 3 min at 98 °C, followed by 30 cycles consisting of 10 s at 98 °C, 20 s at 65 °C, and 20 s at 72 °C for all amplicons. The Ct values obtained from qPCR were utilized in the construction of a PCN standard curve, which yields a linear equation data formula defined as y = Ax + B. In this equation, y denotes the Ct values, while x represents the logarithm of the copies number (log copies/μL).

#### PCN determination using absolute quantification in qPCR

Simultaneous qPCR amplifications were conducted on the total DNA samples obtained from two distinct cultures of *Escherichia coli* DH5α carrying the pBR322, as well as the sample pD451-SR_Taqpol. The process of absolute quantification involves the precise determination of the copy concentration of a target gene by establishing a relationship between the CT value and a standard curve^51,52^. The calculation of PCN is derived from the formula proposed by Plotka et al.^24^.

#### Expression of Taq polymerase in *E. coli* system using autoinduction method

Terrific Broth (TB) supplemented with 30 ppm kanamycin was used as preculture medium for the cultivation of *E. coli* BL21 Star™ (DE3) (Invitrogen, USA) containing the plasmid pD451-SR_Taqpol and was then incubated at a temperature of 37 °C with consistent shaking at 160 rpm for overnight. A total of 1% (v/v) pre-culture was added to 30 mL of chemically defined media containing 30 ppm kanamycin. The chemically defined medium was prepared following the methodology outlined by Nugraha et al.^17^. The culture was subsequently grown under agitation at 165 rpm at a temperature of 37 °C. Following a 24-h period, the culture underwent centrifugation at 6000 × g for 6 min at a temperature of 4 °C in order to isolate the cell pellet from the medium. The cellular pellet was subsequently reconstituted by adding Tris–HCl buffer (25 mM, pH 8.0) supplemented with 1 mM PMSF. To generate a crude enzyme extract, the resuspended cell underwent sonication utilizing an ultrasound probe with an amplitude of around 30% while being placed on ice. This procedure was iterated twice. The crude recombinant protein was subsequently isolated from the cellular debris using centrifugation at 18,000 × g at a temperature of 4 °C for 15 min. Recombinant Taq polymerase expression was assessed on a 10% gel using sodium dodecyl sulfate polyacrylamide gel electrophoresis (SDS-PAGE) according to the Laemmli method^53^.

#### Optimization of carbon source concentration for the optimum yield of Taq polymerase

To generate the highest possible yield of recombinant Taq polymerase, the amount of carbon supplied in the chemically defined medium was optimized. The medium was supplemented with lactose, glucose, and glycerol as the carbon sources. For optimization purpose, the levels of lactose, glycerol, and glucose were modified, and the impact of each carbon sources on the production of recombinant Taq polymerase was examined. Prior to optimization, the medium had standard concentrations of glucose, lactose, and glycerol at 0.05, 0.5, and 0.9%, respectively. The progressive optimization procedure was employed to achieve the best yield of recombinant protein, first with glucose and subsequently advancing to lactose and glycerol. The glucose concentrations were systematically varied (0, 0.025, 0.05, 0.075, 0.1, and 0.15%), whereas the concentrations of lactose and glycerol were established at 0.5 and 0.9%, respectively. In the subsequent attempt to optimize the carbon supply, the lactose content from 0, 0.25, 0.5, 0.75, 1, and 1.5% were used in the medium, while the glucose concentration that yielded the highest ratio of recombinant Taq polymerase was employed, and glycerol was consistently administered at a fixed quantity (0.9%). Furthermore, the concentration of glycerol was manipulated with a variation of 0, 0.3, 0.6, 0.9, 1.2, and 1.5%, respectively, in order to enhance the Taq polymerase yield. During this stage, the optimal concentrations of glucose and lactose chosen based on previous findings were utilized. In each iteration of the optimization process, the qualitative analysis of the recombinant Taq polymerase production was conducted following a 24-h incubation period. The quantification of soluble Taq polymerase was performed using the gel analyzer program, imageJ (USA).

#### Optimization fermentation condition in a 5 L fermentor

After achieving the ideal carbon source composition for Taq polymerase expression, it was further applied in a 5 L fermentor to facilitate the synthesis of Taq polymerase. Further efforts were focused on optimizing the conditions of agitation, aeration, and concentration of inoculant. A 5 L of New Brunswick™ BioFlo^®^ 120 Bioreactor equipped with a Rushton blade (Eppendorf SE, German) with a working volume of 3 L was used to conduct the fermentation process.

For preculture, a colony was introduced into a 150 mL LB medium supplemented with 0.4% glucose and 30 parts per million (ppm) kanamycin using a 500 mL Erlenmeyer flask. The culture was subjected to incubation at 37 °C and a rotational speed of 165 rpm, facilitating its rapid growth. The culture that had undergone overnight incubation was subsequently added at a concentration of 5% (v/v) to a 3 L volume of LB broth supplemented with 30 ppm kanamycin (Sigma-Aldrich, USA). The experimental conditions involved maintaining the culture at a temperature of 37 °C, observing agitation levels ranging from 200 to 400 rpm, and ensuring a constant aeration of 3 vvm. The culture was thereafter subjected to incubation for a period of 24 h, during which sampling was performed at 2-h intervals. Following the centrifugation process, the cell was subsequently suspended in a Tris–HCl buffer with a pH of 8 and a concentration of 25 mM. The crude protein was subsequently recovered from the suspension using sonication. Subsequently, the crude protein underwent electrophoresis on a 10% acrylamide gel and was analyzed using ImageJ (USA) software to quantify the yield of Taq polymerase. Once the desired degree of agitation was attained, the optimal aeration condition was subsequently established. The initial stages were carried out using the same methodology as the prior stages. The fermentation procedure was conducted at a temperature of 37 °C, employing appropriate agitation previously obtained and aeration levels within the range of 2–4 vvm. The cultures were allowed to undergo incubation for a period of 24 h. A volume of 20 mL of the sample was continuously collected at two-hour intervals for further analysis. In accordance with the aforementioned protocols, every cell suspension underwent identical treatment, and the crude protein was subjected to analysis using ImageJ software (USA). The final parameter that was optimized was the initial concentration of the inoculant. Through the implementation of optimal aeration and agitation conditions, a preculture solution with a concentration ranging from 5 to 10% was injected into the culture. The culture was then incubated at a temperature of 37 °C for a duration of 24 h. Sampling was carried out using the same methodology as the preceding phase. An analysis of the expression level of Taq polymerase was conducted using ImageJ (USA).

#### Determination of growth rate

Colony forming unit per mL (CFU/mL) was determined using serial dilutions on LB agar supplemented with 30 ppm kanamycin. Previous to cell counting, the samples were subjected to incubation at a temperature of 37 °C for a period of 16 h. Plates were employed to culture samples in two separate replications. The determination of the maximal specific growth rate (μmax/h) was conducted by the application of linear regression analysis on plots depicting the natural logarithm of the biomass (lnX) as a function of time (t).

#### Purification of Taq polymerase

Once optimal conditions were reached, fermentation carried out, and the cell was harvested to produce a crude Taq-pol. Cell harvesting and crude enzyme extraction were done according to the aforementioned protocols. Following this, the purification process was carried out utilizing HisTrap™ HP column on the AKTA Prime Plus (Cytiva, USA). The HisTrap™ HP column was equilibrated using a binding buffer consisting of 20 mM sodium phosphate, and 20 mM imidazole, 500 mM NaCl, with a pH of 7.4. The equilibration process was carried out at a flow rate of 3 mL min^−1^. Afterwards, the crude extract was loaded onto the column. In order to elute the bound protein, a flow rate of 1 mL min^−1^ was employed in an elution buffer solution consisting of 20 mM sodium phosphate, 500 mM NaCl, and a pH of 7.4. The elution buffer was prepared with a linear gradient of 20–500 mM imidazole. In order to eliminate imidazole, the Taq polymerase fractions that had been purified were combined and subjected to two rounds of dialysis using a 25 mM Tris–HCl buffer with a pH of 8.0. The initial dialysis procedure was performed overnight at a temperature of 4 °C, followed by a subsequent 6-h dialysis session at the identical temperature. A 10% acrylamide gel was utilized to investigate fractions that exhibited the high concentration of Taq polymerase based on the evaluation by AKTA system. The determination of the amount of Taq polymerase was conducted via the Bicinchoninic Acid (BCA) method, wherein bovine serum albumin (BSA) was employed as standard.

#### Western blotting

The SuperSignal West HisProbe kit (Thermoscientific) was utilized to conduct the Western blot analysis. In summary, before transferring the purified Taq polymerase onto a nitrocellulose membrane, it was introduced onto a 10% acrylamide gel. The purified fraction transfer onto a nitrocellulose membrane was conducted using the Mini ProteanVR II trans blot unit (Bio-Rad, USA) and coated with BSA/TBST for a duration of 1 h, while being agitated. Upon blocking the membrane, it was rinsed with 2 mL of TBST for a duration of 10 min each. Following drying, the sample was subjected to incubation with HisProbe-HRP solution for 1 h while being agitated, and subsequently rinsed with 15 mL TBST for four time. The recombinant protein was detected by introducing the KPL TMB (3,30,5,50-tetramethylbenzidine) Peroxidase substrate.

#### Determination the activity of Taq polymerase

Quantitative measurement of the activity of the purified Taq polymerase was conducted using q PCR (Bio-Rad, USA) technique. Initially, a freshly produced qPCR reaction mixture was prepared using the EvaEZ™ Fluorometric Polymerase Activity Assay Kit (Biotium, USA) in accordance with the instructions provided by the manufacturer. A qualitative methodology was employed to assess the activity of Taq polymerase by the amplification of 16S rRNA gene from five bacterial isolates. In accordance with the manufacturer’s instructions, genomic DNA from each of the isolates of bacteria was isolated using the Quick-DNA Miniprep Kit (ZYMO RESEARCH). The synthesis of a gene encoding _D_-allulose 3-epimerasewas (DAEase) was carried out through PCR, utilizing 1 × buffer, oligonucleotide primers (200 nM), 3.5 mM MgCl2, and DNA template (1 ng). The quantity of purified Taq-pol utilized for DNA amplification ranged from 0.15 to 1.25 U, while 0.625 U of Amplitaq Gold 360 DNA polymerase (Applied Biosystem, ThermoFisher, USA) served as a control. Each PCR reaction started with an initial denaturation step at a temperature of 95 °C for a duration of 5 min. This was continued by 35 cycles of denaturation steps at 95 °C for 30 s, an annealing step at 64 °C for 40 s, an extension step at 72 °C for 2 min, and finally a final extension step at 72 °C for 7 min. The PCR results were subjected to electrophoresis on a 0.8% agarose gel in Tris-Boric EDTA (TBE) buffer using an electrophoratic apparatus. To assess the efficacy of PCR amplification, the visualization of DNA bands was performed using SYBR green and a UV Transilluminator technique.

## Supplementary Information

Supplementary Information.

## Data Availability

This article contains all of the data collected or analysed during the course of the study.

## References

[1] Wang, G. et al. Thermophilic nucleic acid polymerases and their application in xenobiology. *Int. J. Mol. Sci.* **23**, 14969. 10.3390/ijms232314969 (2022). 36499296 10.3390/ijms232314969 PMC9738464

[2] Lawyer, F. C. et al. High-level expression, purification, and enzymatic characterization of full-length *Thermus aquaticus* DNA polymerase and a truncated form deficient in 5′ to 3′ exonuclease activity. *Genome Res.* **2**, 275–287 (1993). 10.1101/gr.2.4.275 8324500

[3] Aschenbrenner, J. & Marx, A. DNA polymerases and biotechnological applications. *Curr. Opin. Biotechnol.* **48**, 187–195. 10.1016/j.copbio.2017.04.005 (2017). 28618333 10.1016/j.copbio.2017.04.005

[4] McInerney, P., Adams, P. & Hadi, M. Z. Error rate comparison during polymerase chain reaction by DNA polymerase. *Mol. Biol. Int.* **2014**, 287430 (2014). 25197572 10.1155/2014/287430 PMC4150459

[5] Ishino, S. & Ishino, Y. DNA polymerases as useful reagents for biotechnology â€“ the history of developmental research in the field. *Front. Microbiol.* **5**, 465 (2014). 25221550 10.3389/fmicb.2014.00465 PMC4148896

[6] Yamagami, T., Ishino, S., Kawarabayasi, Y. & Ishino, Y. Mutant Taq DNA polymerases with improved elongation ability as a useful reagent for genetic engineering. *Front. Microbiol.* **5**, 461 (2014). 25232352 10.3389/fmicb.2014.00461 PMC4153296

[7] Korolev, S., Nayal, M., Barnes, W. M., Di Cera, E. & Waksman, G. Crystal structure of the large fragment of *Thermus aquaticus* DNA polymerase I at 2.5-Å resolution: Structural basis for thermostability. *Proc. Natl. Acad. Sci. U. S. A.* **92**, 9264–9268 (1995). 7568114 10.1073/pnas.92.20.9264 PMC40965

[8] Villbrandt, B., Sobek, H., Frey, B. & Schomburg, D. Domain exchange: Chimeras of *Thermus aquaticus* DNA polymerase, *Escherichia coli* DNA polymerase I and *Thermotoga neapolitana* DNA polymerase. *Protein Eng.* **13**, 645–654 (2000). 11054459 10.1093/protein/13.9.645

[9] Yang, S. W., Astatke, M., Potter, J. & Chatterjee, D. K. Mutant *Thermotoga neapolitana* DNA polymerase I: Altered catalytic properties for non-templated nucleotide addition and incorporation of correct nucleotides. *Nucleic Acids Res.* **30**, 4314–4320. 10.1093/nar/gkf547 (2002). 12364611 10.1093/nar/gkf547 PMC140547

[10] Chen, S. et al. A simple and efficient method for extraction of Taq DNA polymerase. *Electron. J. Biotechnol.* **18**, 355–358 (2015).

[11] Pluthero, F. G. Rapid purification of high-activity Taq DNA polymerase. *Nucleic Acids Res.* **21**, 4850–4851. 10.1093/nar/21.20.4850 (1993). 8233838 10.1093/nar/21.20.4850 PMC331521

[12] Samman, N., Al-Muhalhil, K. & Nehdi, A. A simple and efficient method for Taq DNA polymerase purification based on heat denaturation and affinity chromatography. *J. King Saud Univ. Sci.* **35**, 102565 (2023).

[13] Laksmi, F. A. et al. High-level expression of codon-optimized Taq DNA polymerase under the control of rhaBAD promoter. *Anal. Biochem.* **692**, 115581 (2024). 38815728 10.1016/j.ab.2024.115581

[14] Studier, F. W. Protein production by auto-induction in high density shaking cultures. *Protein Expr. Purif.* **41**, 207–234 (2005). 15915565 10.1016/j.pep.2005.01.016

[15] Liu, Y.-C., Chang, W.-M. & Lee, C.-Y. Effect of oxygen enrichment aeration on penicillin G acylase production in high cell density culture of recombinant *E. coli*. *Bioprocess Eng.* **21**, 227 (1999).

[16] Shahzadi, I. et al. Scale-up fermentation of *Escherichia coli* for the production of recombinant endoglucanase from *Clostridium thermocellum*. *Sci. Rep.* **11**, 7145 (2021). 33785771 10.1038/s41598-021-86000-z PMC8009960

[17] Nugraha, Y., Laksmi, F. A., Nuryana, I., Helbert, & Khasna, F. N. Production of reverse transcriptase from *Moloney murine* Leukemia virus in *Escherichia col*i expression system. *Prep. Biochem. Biotechnol.* **54**, 1079–1087. 10.1080/10826068.2024.2317311 (2024). 38411149 10.1080/10826068.2024.2317311

[18] Hadi, M. I. et al. An efficient approach for overproduction of DNA polymerase from *Pyrococcus furiosus* using an optimized autoinduction system in *Escherichia coli*. *World J. Microbiol. Biotechnol.* **40**, 324 (2024). 39294482 10.1007/s11274-024-04127-3

[19] Burgos, J. S., Ramírez, C., Tenorio, R., Sastre, I. & Bullido, M. J. Influence of reagents formulation on real-time PCR parameters. *Mol. Cell. Probes* **16**, 257–260 (2002). 12270266 10.1006/mcpr.2002.0419

[20] Klein, D. Quantification using real-time PCR technology: Applications and limitations. *Trends Mol. Med.* **8**, 257–260. 10.1016/S1471-4914(02)02355-9 (2002). 12067606 10.1016/s1471-4914(02)02355-9

[21] Rao, X., Lai, D. & Huang, X. A new method for quantitative real-time polymerase chain reaction data analysis. *J. Comput. Biol.* **20**, 703–711 (2013). 23841653 10.1089/cmb.2012.0279 PMC3762066

[22] Zhang, Y. et al. Evaluation validation of a qPCR curve analysis method and conventional approaches. *BMC Genomics* **22**, 1–12 (2021). 34789146 10.1186/s12864-021-07986-4 PMC8596907

[23] Boone, B. A. & Lotze, M. T. Targeting damage-associated molecular pattern molecules (DAMPs) and DAMP receptors in melanoma. *Methods Mol. Biol.* **1102** (2014). 10.1007/978-1-62703-727-3_29 24258998

[24] Plotka, M., Wozniak, M. & Kaczorowski, T. Quantification of plasmid copy number with single colour droplet digital PCR. *PLoS One* **12**, e0169846 (2017). 28085908 10.1371/journal.pone.0169846 PMC5234771

[25] Son, Y. J., Ryu, A. J., Li, L., Han, N. S. & Jeong, K. J. Development of a high-copy plasmid for enhanced production of recombinant proteins in *Leuconostoc citreum*. *Microb. Cell Fact.* **15**, 1–11 (2016). 26767787 10.1186/s12934-015-0400-8 PMC4714500

[26] Athmaram, T. N. et al. Influence of copy number on the expression levels of pandemic influenza hemagglutinin recombinant protein in methylotrophic yeast *Pichia pastoris*. *Virus Genes* **45**, 440–451 (2012). 22940846 10.1007/s11262-012-0809-7

[27] O’Connell, H. A., Niu, C. & Gilbert, E. S. Enhanced high copy number plasmid maintenance and heterologous protein production in an *Escherichia coli* biofilm. *Biotechnol. Bioeng.* **97**, 439–446 (2007). 17058286 10.1002/bit.21240

[28] Balbás, P. et al. Plasmid vector pBR322 and its special-purpose derivatives—a review. *Gene* **50**, 3–40 (1986). 3034735 10.1016/0378-1119(86)90307-0

[29] Grabherr, R., Nilsson, E., Striedner, G. & Bayer, K. Stabilizing plasmid copy number to improve recombinant protein production. *Biotechnol. Bioeng.* **77**, 142–147 (2002). 11753920 10.1002/bit.10104

[30] Argyropoulos, D. & Lynch, H. C. Recombinant β-glucanase production and plasmid stability of *Bacillus subtilis* in cyclic fed batch culture. *Biotechnol. Tech.* **11**, 187–190 (1997).

[31] Summers, D. K. The kinetics of plasmid loss. *Trends Biotechnol.* **9**, 273–278. 10.1016/0167-7799(91)90089-Z (1991). 1367567 10.1016/0167-7799(91)90089-z

[32] Providenti, M. A., O’Brien, J. M., Ewing, R. J., Paterson, E. S. & Smith, M. L. The copy-number of plasmids and other genetic elements can be determined by SYBR-Green-based quantitative real-time PCR. *J. Microbiol. Methods* **65**, 476–487 (2006). 16216354 10.1016/j.mimet.2005.09.007

[33] Zhong, C. et al. Determination of plasmid copy number reveals the total plasmid DNA amount is greater than the chromosomal DNA amount in *Bacillus thuringiensis* YBT-1520. *PLoS One* **6**, e16025 (2011). 21283584 10.1371/journal.pone.0016025 PMC3026805

[34] Blommel, P. G., Becker, K. J., Duvnjak, P. & Fox, B. G. Enhanced bacterial protein expression during auto-induction obtained by alteration of lac repressor dosage and medium composition. *Biotechnol. Prog.* **23**, 585–598 (2007). 17506520 10.1021/bp070011x PMC2747370

[35] Machado, R. et al. High level expression and facile purification of recombinant silk-elastin-like polymers in auto induction shake flask cultures. *AMB Express* **3**, 1–15 (2013). 23384239 10.1186/2191-0855-3-11 PMC3599559

[36] Lee, S. Y. High cell-density culture of *Escherichia coli*. *Trends Biotechnol.* **14**, 98–105. 10.1016/0167-7799(96)80930-9 (1996). 8867291 10.1016/0167-7799(96)80930-9

[37] Zhang, J., Reddy, J., Buckland, B. & Greasham, R. Toward consistent and productive complex media for industrial fermentations: Studies on yeast extract for a recombinant yeast fermentation process. *Biotechnol. Bioeng.* **82**, 640–652 (2003). 12673763 10.1002/bit.10608

[38] Jacob, F. & Monod, J. Genetic regulatory mechanisms in the synthesis of proteins. *J. Mol. Biol.* **3**, 318–356. 10.1016/S0022-2836(61)80072-7 (1961). 13718526 10.1016/s0022-2836(61)80072-7

[39] Darah, I., Sumathi, G., Jain, K. & Lim, S. H. Influence of agitation speed on tannase production and morphology of *Aspergillus niger* FETL FT3 in submerged fermentation. *Appl. Biochem. Biotechnol.* **165**, 1682–1690 (2011). 21947762 10.1007/s12010-011-9387-8

[40] Potumarthi, R., Subhakar, C. & Jetty, A. Alkaline protease production by submerged fermentation in stirred tank reactor using *Bacillus licheniformis* NCIM-2042: Effect of aeration and agitation regimes. *Biochem. Eng. J.* **34**, 185–192 (2007).

[41] Potumarthi, R., Subhakar, C., Vanajakshi, J. & Jetty, A. Effect of aeration and agitation regimes on lipase production by newly isolated *Rhodotorula mucilaginosa*—MTCC 8737 in stirred tank reactor using molasses as sole production medium. *Appl. Biochem. Biotechnol.* **151**, 700–710 (2008). 18574564 10.1007/s12010-008-8293-1

[42] Mantzouridou, F., Roukas, T. & Kotzekidou, P. Effect of the aeration rate and agitation speed on β-carotene production and morphology of *Blakeslea trispora* in a stirred tank reactor: Mathematical modeling. *Biochem. Eng. J.* **10**, 123–135 (2002).

[43] Giavasis, I., Harvey, L. M. & McNeil, B. The effect of agitation and aeration on the synthesis and molecular weight of gellan in batch cultures of *Sphingomonas paucimobilis*. *Enzyme Microb. Technol.* **38**, 101–108 (2006).

[44] Bandaiphet, C. & Prasertsan, P. Effect of aeration and agitation rates and scale-up on oxygen transfer coefficient, kLa in exopolysaccharide production from *Enterobacter cloacae* WD7. *Carbohydr. Polym.* **66**, 216–228 (2006).

[45] Mishra, M. N., Mohanraj, J., Nisshanthini, S. D. & Bhat, S. High-level expression and purification of DNA and DNase free Taq DNA polymerase. *Asian J. Res. Biochem.*10.9734/ajrb/2018/v2i4607 (2018).

[46] Handayani, C. V. et al. Expression of soluble moloney murine leukemia virus-reverse transcriptase in *Escherichia coli* BL21 star (DE3) using autoinduction system. *Mol. Biol. Rep.* **51**, 1–11 (2024). 10.1007/s11033-024-09583-6 38717629

[47] Sopitthummakhun, K. et al. Plasmodium serine hydroxymethyltransferase as a potential anti-malarial target: Inhibition studies using improved methods for enzyme production and assay. *Malar. J.* **11**, 1–12 (2012). 22691309 10.1186/1475-2875-11-194 PMC3502260

[48] Wang, Z. et al. Engineering Escherichia coli for cost-effective production of medium-chain fatty acids from soy whey using an optimized galactose-based autoinduction system. *Bioresour. Technol.* **393**, 130145 (2024). 38042430 10.1016/j.biortech.2023.130145

[49] Vogels, C. B. F. et al. Analytical sensitivity and efficiency comparisons of SARS-CoV-2 RT–qPCR primer–probe sets. *Nat. Microbiol.* **5**, 1299–1305 (2020). 32651556 10.1038/s41564-020-0761-6 PMC9241364

[50] Whelan, J. A., Russell, N. B. & Whelan, M. A. A method for the absolute quantification of cDNA using real-time PCR. *J. Immunol. Methods* **278**, 261–269 (2003). 12957413 10.1016/s0022-1759(03)00223-0

[51] Yu, Y., Lee, C., Kim, J. & Hwang, S. Group-specific primer and probe sets to detect methanogenic communities using quantitative real-time polymerase chain reaction. *Biotechnol. Bioeng.* **89**, 670–679 (2005). 15696537 10.1002/bit.20347

[52] Lee, C., Kim, J., Shin, S. G. & Hwang, S. Absolute and relative QPCR quantification of plasmid copy number in *Escherichia coli*. *J. Biotechnol.* **123**, 273–280 (2006). 16388869 10.1016/j.jbiotec.2005.11.014

[53] Laemmli, U. K. Cleavage of structural proteins during the assembly of the head of bacteriophage T4. *Nature* **227**, 680–685 (1970). 5432063 10.1038/227680a0

