# Supplementary material for: A robust strategy for overexpression of DNA polymerase from Thermus aquaticus using an IPTG-independent autoinduction system in a benchtop bioreactor
Source: Sci Rep. 2025 Feb 18;15:5891. doi: 10.1038/s41598-025-89902-4 (PMC11836315; doi:10.1038/s41598-025-89902-4)

**Supplementary Figure S1.** The standard curve used for absolute quantification of plasmid copy number.


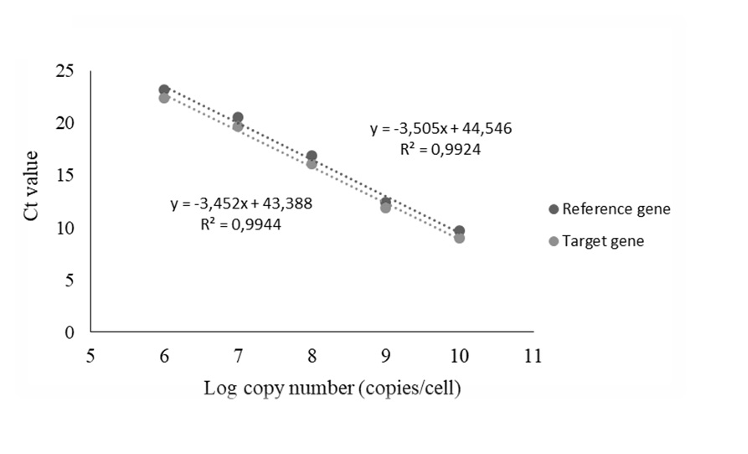

Supplement: Supplementary file 1 — Supplementary Information. [file 41598_2025_89902_MOESM1_ESM.docx]
